# *PIK3R1Met326Ile* germline mutation correlates with cysteine-rich protein 61 expression and poor prognosis in glioblastoma

**DOI:** 10.1038/s41598-017-07745-0

**Published:** 2017-08-07

**Authors:** Yoshihiro Otani, Joji Ishida, Kazuhiko Kurozumi, Tetsuo Oka, Toshihiko Shimizu, Yusuke Tomita, Yasuhiko Hattori, Atsuhito Uneda, Yuji Matsumoto, Hiroyuki Michiue, Shuta Tomida, Takehiro Matsubara, Tomotsugu Ichikawa, Isao Date

**Affiliations:** 1 Department of Neurological Surgery, Okayama University Graduate School of Medicine, Dentistry, and Pharmaceutical Sciences, Okayama, Japan; 2 Department of Physiology, Okayama University Graduate School of Medicine, Dentistry, and Pharmaceutical Sciences, Okayama, Japan; 3 Okayama University Hospital Biobank, Okayama University Hospital, Okayama, Japan; 4 Department of Biobank, Graduate School of Medicine, Dentistry and Pharmaceutical Sciences, Okayama University, Okayama, Japan

## Abstract

Despite therapeutic advances, glioblastoma represents a lethal brain tumor. Recently, research to identify prognostic markers for glioblastoma has intensified. Our previous study demonstrated that median progression-free survival (PFS) and overall survival (OS) of patients with high cysteine-rich protein 61 (CCN1) expression was significantly shorter than that of patients with low CCN1 expression. To understand the molecular mechanisms that regulate CCN1 expression, we examined 147 tumour samples from 80 patients with glioblastoma and 67 patients with lower grade glioma. Next-generation and Sanger sequencing showed that *PIK3R1Met326Ile* was more frequent in the CCN1 high expression group (10/37 cases, 27.0%) than the CCN1 low expression group (3/38 cases, 7.9%) in glioblastoma. This mutation was also detected in corresponding blood samples. In multivariate analysis, high CCN1 expression and *PIK3R1Met326Ile* in glioblastoma patients were prognostic factors for OS [HR = 2.488 (1.298–4.769), *p* = 0.006] and [HR = 2.089 (1.020–4.277), p = 0.0439], respectively. Thus, the *PIK3R1Met326Ile* germline appears to be correlated with CCN1 expression and poor prognosis in glioblastoma.

## Introduction

Glioblastoma is the most common type of primary malignant brain tumor in adults, with a median survival time of 14 to 16 months, even after the most aggressive treatment, including maximum resection, radiation and chemotherapy^1–3^. Recently, the poor prognosis of glioblastoma patients has been linked to intratumoral genetic heterogeneity^4^, and many researchers have investigated the genetic and epigenetic alterations of glioblastoma^5, 6^.

Concurrently, several prognostic molecular biomarkers for glioblastoma have been reported. The cellular DNA repair protein, O^6^-methylguanine-DNA methyltransferase (MGMT), inhibits the cross-linking of double-stranded DNA by removing alkylation lesions, determines the effect of temozolomide, and is independently associated with overall survival (OS) of malignant glioma patients^7, 8^. Somatic mutations in the metabolic enzyme isocitrate dehydrogenase (IDH) have also been found in glioma, and glioblastoma patients with wild-type IDH had a poorer prognosis than those with mutant IDH^9, 10^. In previous research, high expression of cysteine-rich protein 61 (CCN1; also known as CYR61) correlated with a poorer prognosis in glioblastoma patients^11^.

CCN1, a 42-kDa, secreted, heparin-binding protein, is one of the prototypical members of the CCN family of matricellular proteins^12^. It was first identified as a growth factor-inducible immediate-early gene, which promoted proliferative responses to growth factors through its interaction with cell surface integrins^13, 14^. In many cancers, CCN1 may play an important role in tumor progression, and expression levels of CCN1 are related to patient prognosis^15, 16^. The mechanism of CCN1 expression and genetic alterations in malignant tumors have not been well-characterized.

Somatic mutations in *PIK3R1* are associated with several cancers types, in particular, endometrial carcinoma (33.8%), metastatic prostate adenocarcinoma (11.5%), and colorectal adenocarcinoma (9.7%)^17^. According to The Cancer Genome Atlas (TCGA) database, PIK3R1 is the 11^th^ most commonly mutated gene across 4,429 tumors covering 20 diseases^18^. With respect to glioblastoma, *PIK3R1* mutations represent one of the most common genetic aberrations, and the phosphoinositide 3-kinase (PI3K) pathway is one of the most frequently targeted signaling pathways for therapeutic strategies. However, in TCGA, somatic mutations were analyzed, but germline mutations were not^6^.

Some germline mutations are known to participate in different types of familial glioma. Germline mutations in *p53* may give rise to Li-Fraumeni syndrome, germline mutations in the mismatch DNA repair genes *MLH1* and *PMS2* are implicated in Turcot syndrome, and individuals with germline mutations in the *NF1* or *NF2* gene are susceptible to neurofibromatosis, which progresses to glioma^19^. However, the relationship between germline mutations and tumorigenesis or prognosis has not been fully elucidated.

In this study, the focus was to identify genetic alterations that correlate with CCN1 expression. The results demonstrated that a germline mutation in *PIK3R1* (p85α, the regulatory subunit of PI3K) occurred at a higher rate in patients with high CCN1 expression.

## Results

### The expression level of CCN1 in immunohistochemical staining correlated with cleaved CCN1 expression in western blotting

**Figure 1:**
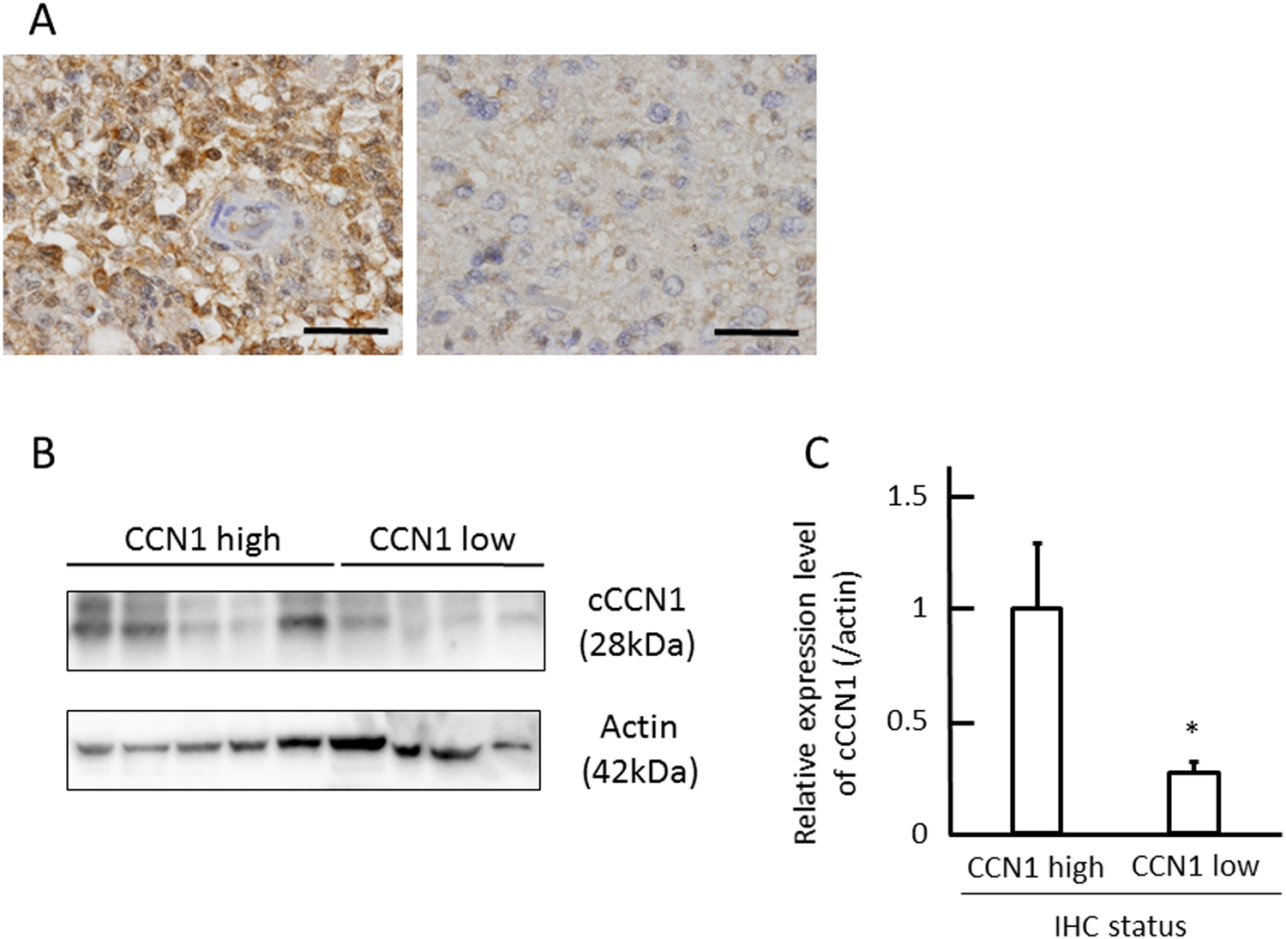
The relationship between CCN1 in immunohistochemical staining and cleaved CCN1 (cCCN1) in western blotting. (**A**) Representative immunohistochemical staining of CCN1 expression in glioblastoma multiforme patients. The photomicrographs show Left; CCN1 high, Right; CCN1 low. Scale bar = 50 μm. (**B**,**C**) Expression level of the NH2-terminal fragment of CCN1 protein (cCCN1, 28 kDa) determined by western blotting compared with immunohistochemical staining of CCN1 performed on formalin-fixed paraffin-embedded tissues from 16 corresponding tumors. Correlation was assessed using the Mann-Whitney U test with *p* < 0.05 considered as significant. Data are shown as the mean ± SEM. Full-length blots are presented in Supplementary Figure S2.

To identify genetic alterations that correlate with CCN1 expression, we first examined CCN1 levels in glioblastoma cases. A total of 147 tumour samples from 80 patients with glioblastoma and 67 patients with lower grade glioma were analysed and we classified cases based on high or low CCN1 expression level (as described in Methods). Representative high and low CCN1 immunohistochemical stainings are shown in Fig. 1A. Subsequently, the expression level of cleaved CCN1 (cCCN1), which is the truncated form of CCN1 and associated with biological activity in cancer^20, 21^, was analyzed. Western blotting showed that the NH2-terminal fragment of CCN1 protein was more highly expressed in the group with high CCN1 compared with low CCN1 in immunohistochemical staining (Fig. 1B and C, *p* < 0.05).

### Genetic alterations in high and low CCN1 glioblastoma

**Figure 2:**
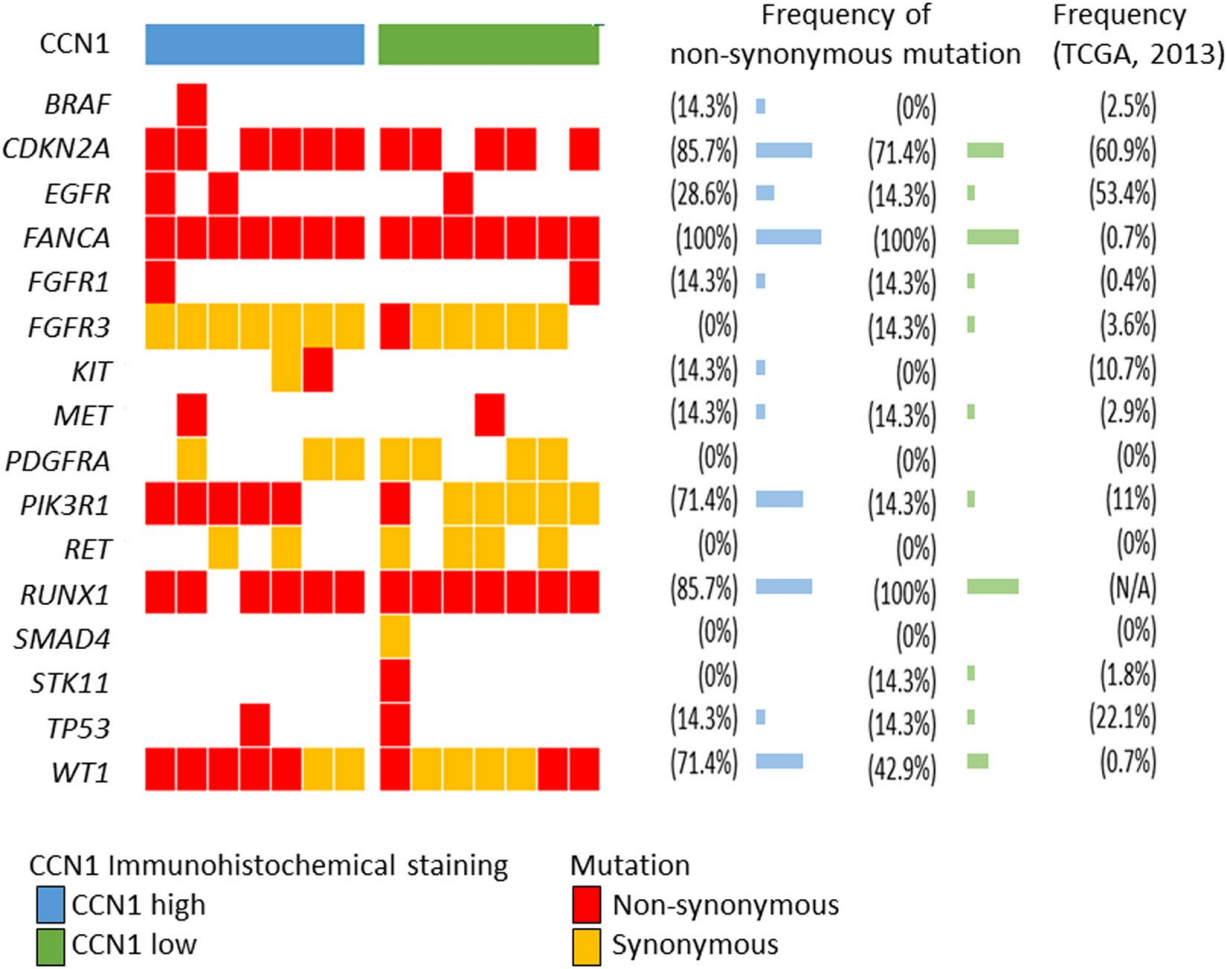
Mutations in high and low CCN1 expression glioblastoma. Left; Specific genes harboring nonsynonymous and synonymous mutations are shown. Middle; The frequency of nonsynonymous mutations in each of the listed genes in both CCN1 groups. Right; The frequency of genetic alterations in each of the listed genes in The Cancer Genome Atlas (TCGA) dataset (2013).

The genetic alterations related to CCN1 expression were investigated using a HaloPlex Cancer Research Panel, which targets Catalogue of Somatic Mutations in Cancer (COSMIC) mutations within 47 genes known to be associated with cancer. Using immunohistochemical staining, 7 samples in the CCN1 high expression group, 7 samples in the CCN1 low expression group, and 1 normal brain tissue were evaluated (Fig. 2). All 14 glioblastoma samples were wild-type *IDH*. The total number of nonsynonymous amino acid alterations was 47 and 38 in the CCN1 high and low expression groups, respectively (F = 0.450, t = 0.739, *p* = 0.474, Student’s t-test). In contrast, the number of specific *PIK3R1* mutations (chromosome 5, 67588148 G- > A, Met326Ile) tended to be higher in the CCN1 high expression group (4/7 cases, 57.1%) compared with the CCN1 low expression group (1/7 cases, 14.3%) (*p* = 0.266, Fisher’s exact test) (Table 1). Sanger sequencing of corresponding blood samples demonstrated that the *PIK3R1Met326Ile* mutation was detected in patients with *PIK3R1Met326Ile*-mutant glioma specimens (Table 1). Copy-number variation analysis showed no significant difference in gain (14 and 28 genes in the CCN1 high and low expression group, respectively, F = 17.455, t = 1.173, *p* = 0.263, Student’s t-test) or loss (86 and 81 genes in the CCN1 high and low expression group, respectively, F = 0.05, t = 0.374, *p* = 0.715, Student’s t-test).

**Table 1:** The results of HaloPlex Cancer Research Panel analysis of glioma specimens and Sanger sequencing of corresponding blood samples for the PIK3R1 germline mutation

Sample	CCN1 IHC	HaloPlex Cancer Research Panel of glioma specimens		Sanger sequencing of blood
		PIK3R1 mutation	*PIK3R1 Met326Ile* Allele Frequency	*PIK3R1 Met326Ile*
1	High	*PIK3R1 Met326Ile*	0.494	Positive
2	High	*PIK3R1 Met326Ile*	0.558	Positive
3	High	*PIK3R1 Met326Ile*	0.483	Positive
4	High	*PIK3R1 Met326Ile*	0.482	N/A
5	High	*PIK3R1 Pro205Leu*		Negative
6	High	Wild type		Negative
7	High	Wild type		Negative
8	Low	*PIK3R1 Met326Ile*	0.49	Positive
9	Low	Wild type		Negative
10	Low	Wild type		Negative
11	Low	Wild type		Negative
12	Low	Wild type		Negative
13	Low	Wild type		Negative
14	Low	Wild type		Negative

IHC = immunohistochemistry; N/A = not available.

### The frequency of *PIK3R1Met326Ile* was higher in the CCN1 high expression group

**Figure 3:**
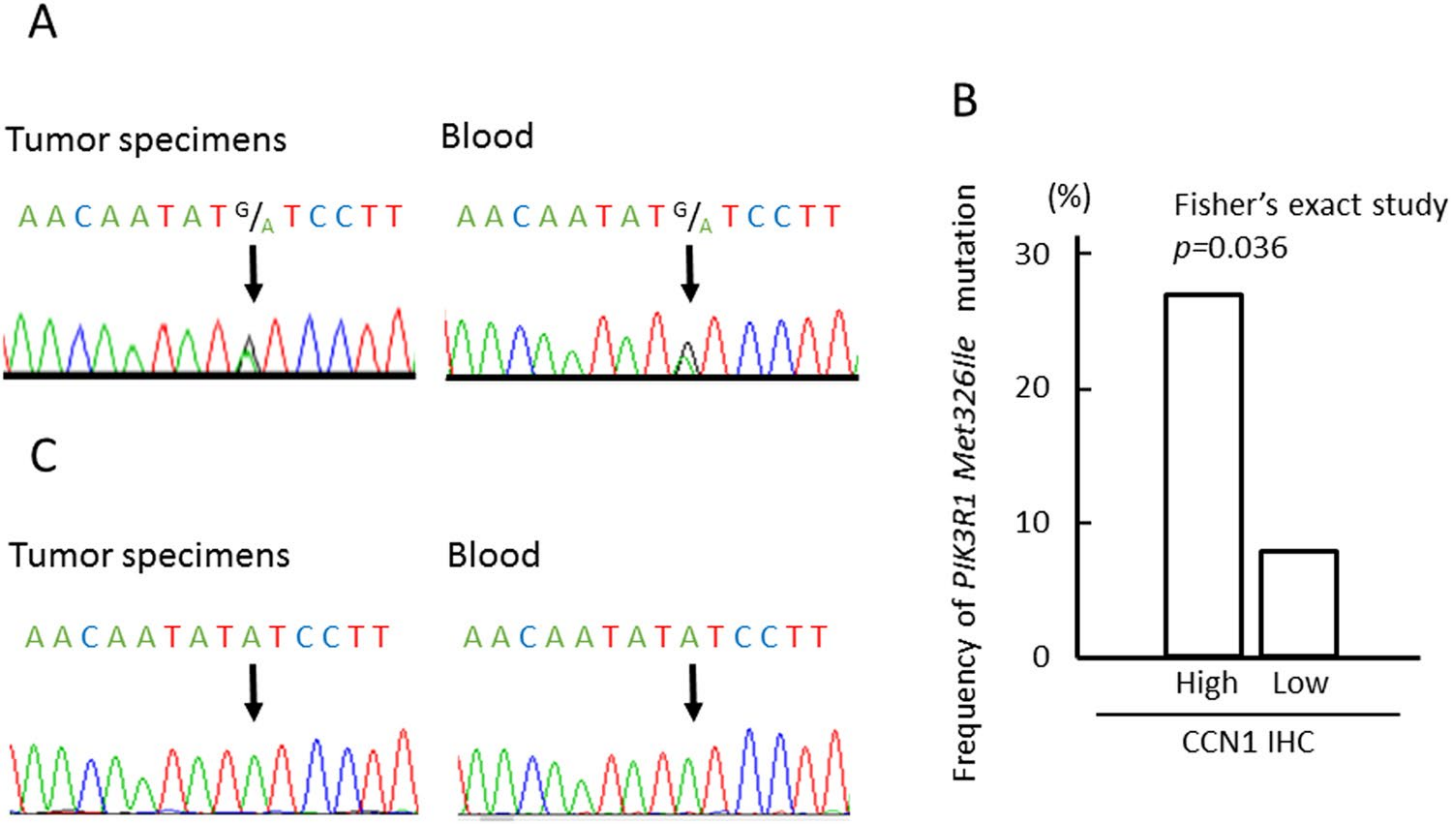
*PIK3R1Met326Ile* mutations in glioblastoma patients. (**A**) Electropherogram of patients with heterozygous mutation of *PIK3R1Met326Ile*. (**B**) The frequency of *PIK3R1Met326Ile* mutations was significantly higher in the CCN1 high expression group compared with the CCN1 low expression group. Statistical significance was calculated by Fisher’s exact test. (**C**) Electropherogram of patients with homozygous mutation of *PIK3R1Met326Ile*.

Deep sequencing data revealed that the frequency of the *PIK3R1Met326Ile* mutation tended to be higher in the CCN1 high expression group on immunohistochemical staining. To examine the relevance of the *PIK3R1Met326Ile* mutation and CCN1 expression, *PIK3R1Met326Ile* mutations were analyzed in additional glioblastoma specimens and lower grade glioma specimens (Tables 2 and 3, and Supplementary Table S1). We investigated 80 glioblastoma specimens and 67 lower grade glioma specimens, including 39 anaplastic astrocytoma, 8 diffuse astrocytoma, 14 anaplastic oligodendroglioma, and 6 oligodendroglioma samples. Five paediatric glioblastoma patients and 7 paediatric lower grade glioma were excluded from the cohorts, because recent studies showed that children and adult glioma represent molecularly distinct entities with differing biological backgrounds^22–25^. The overall frequencies of the *PIK3R1Met326Ile* mutation in adult glioblastoma patients and adult lower grade glioma patients were 17.3% (13/75 cases) and 16.7% (10/60 cases), respectively, and conforms to Hardy-Weinberg proportions. The frequency of *PIK3R1Met326Ile* in glioblastoma was significantly higher in the CCN1 high expression group (10/37 cases, 27.0%) than the CCN1 low expression group (3/38 cases, 7.9%) (*p* = 0.036, Fisher’s exact test) (Fig. 3A and B). In all except 1 case of glioblastoma, *PIK3R1Met326Ile* mutations were heterozygotic (Fig. 3C). In lower grade glioma, the CCN1 high expression group tended to have a high frequency of *PIK3R1Met326Ile*, but it was not statistically significant (Table 3 and Supplementary Table S1; astrocytoma, *p* = 0.2488; oligodendroglioma, *p* = 0.3158, Fisher’s exact test).

**Table 2:** Clinical and genetic characteristics of the glioblastoma study cohort.

Characteristic	Total cases (N = 75)	CCN1 high (N = 37)	CCN1 low (N = 38)
Age - year			
Median	60	66	56
Range	19–81	25–76	19–81
Sex - no.(%)			
Male	39 (52.0)	21 (56.8)	18 (47.4)
Female	36 (48.0)	16 (43.2)	20 (52.6)
Surgical status – no.(%)			
Biopsy or partial resection	41 (54.7)	28 (75.7)	13 (34.2)
Gross total resection	34 (35.3)	9 (24.3)	25 (65.8)
Radiotherapy after 1^st^ operation – no.(%)			
Standard radiotherapy	68 (90.7)	34 (91.9)	34 (89.5)
BNCT	4 (5.3)	1 (2.7)	3 (7.9)
None	3 (4.0)	2 (5.4)	1 (2.6)
Chemotherapy after 1^st^ operation– no.(%)			
Temozolomide only	63 (84.0)	32 (86.5)	31 (81.6)
Temozolomide + others	6 (8.0)	3 (8.1)	3 (7.9)
Others	4 (5.3)	1 (2.7)	3 (7.9)
None	2 (2.7)	1 (2.7)	1 (2.6)
CCN1 expression– no.(%)			
High	37 (49.3)		
Low	38 (50.7)		
*PIK3R1Met326Ile* mutation – no.(%)			
Positive	13 (17.3)	10 (27.0)	3 (7.9)
Negative	62 (82.7)	27 (73.0)	35 (92.1)

SRS = stereotactic radiosurgery; BNCT = boron neutron capture therapy.

**Table 3:** Clinical and genetic characteristics of the astrocytoma study cohort.

Characteristic	Total cases (N = 41)	CCN1 high (N = 22)	CCN1 low (N = 19)
Age - year			
Median	45	53	40
Range	19–82	19–82	20–77
Sex - no.(%)			
Male	24 (59.0)	13 (59)	11 (58)
Female	17 (41.0)	9 (41)	8 (42)
Grade – no.(%)			
Grade II	6 (14.6)	5 (22.7)	1 (5.2)
Grade III	35 (85.4)	17 (77.3)	18 (94.8)
Surgical status – no.(%)*			
Biopsy or partial resection	29 (72.5)	17 (81)	12 (63.2)
Gross total resection	11 (27.5)	4 (19)	7 (36.8)
CCN1 expression– no.(%)			
High	22 (53.7)		
Low	19 (46.3)		
*PIK3R1Met326Ile* mutation – no.(%)			
Positive	8 (19.5)	6 (27.3)	2 (10.5)
Negative	33 (80.5)	16 (72.7)	17 (89.5)

*We could not investigate the extension of resection of one patient.

### Glioblastoma patients with *PIK3R1Met326Ile* had poor PFS and OS

**Figure 4:**
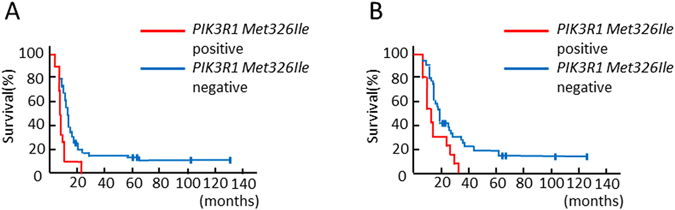
Kaplan-Meier survival curves for patients with and without *PIK3R1Met326Ile* mutations. (**A**) Progression-free survival of patients with *PIK3R1Met326Ile* was significantly shorter than that of patients without *PIK3R1Met326Ile* (*p* = 0.0407). (**B**) Overall survival of patients with *PIK3R1Met326Ile* was significantly shorter than that of patients without *PIK3R1Met326Ile* (*p* = 0.0125).

To investigate the relationship between clinical characteristics and prognosis, PFS and OS curves relative to *PIK3R1Met326Ile* mutations were obtained using the Kaplan-Meier method. The survival curves were compared using a log-rank test based on age, sex, surgical status, CCN1 expression and *PIK3R1* mutational status. The results showed that gross total resection, high CCN1 expression and *PIK3R1Met326Ile* (Fig. 4A) were significantly associated with PFS (*p* = 0.0138, 0.0013 and 0.0407, respectively). Furthermore, gross total resection, high CCN1 expression and *PIK3R1Met326Ile* (Fig. 4B) were significantly associated with OS (*p* = 0.0163, 0.0008 and 0.0125, respectively) (Table 4).

**Table 4:** Univariate analysis of prognostic factors for progression-free survival and overall survival in glioblastoma patients.

	No. of patients (%)	*p* value	
		PFS	OS
Age (years)			
≦65	39 (52.0)	0.9047	0.8401
<65	36 (48.0)		
Sex			
Male	39 (52.0)	0.9047	0.8401
Female	36 (48.0)		
Surgical status			
Biopsy or partial resection	41 (54.7)	0.0138	0.0163
Gross total resection	34 (45.3)		
CCN1 expression			
High	37 (49.3)	0.0013	0.0008
Low	38 (50.7)		
*PIK3R1Met326Ile*			
Positive	13 (17.3)	0.0407	0.0125
Negative	62 (82.7)		

PFS = progression free survival; OS = overall survival.

In multivariate analysis, high CCN1 was a prognostic factor for PFS [hazard ratio (HR) = 2.109 (1.105–4.025), *p* = 0.0236] and OS [HR = 2.488 (1.298–4.769), *p* = 0.0060], while the *PIK3R1Met326Ile* mutation was a prognostic factor for OS [HR = 2.089 (1.020–4.277), *p* = 0.0439] (Table 5).

**Table 5:** Multivariate analysis of prognostic factors for progression-free survival and overall survival in glioblastoma patients.

Variable	Hazard Ration	95% CI	*P* value
Progression-free survival			
Gross total resection	1.316	0.690 to 2.512	0.4043
CCN1 high expression	2.109	1.105 to 4.025	0.0236
* PIK3R1Met326Ile* positive	1.431	0.703 to 2.913	0.3234
Overall survival			
Gross total resection	1.321	0.672 to 2.599	0.4198
CCN1 high expression	2.488	1.298 to 4.769	0.0060
* PIK3R1Met326Ile* positive	2.089	1.020 to 4.277	0.0439

In contrast, among lower grade patients, only age was a prognostic factor and neither CCN1 nor *PIK3R1Met326Ile* was a prognostic factor (Supplementary Tables S2 and S3).

### Glioblastoma patients with high CCN1 expression showed high Akt phosphorylation (p-Akt)

**Figure 5:**
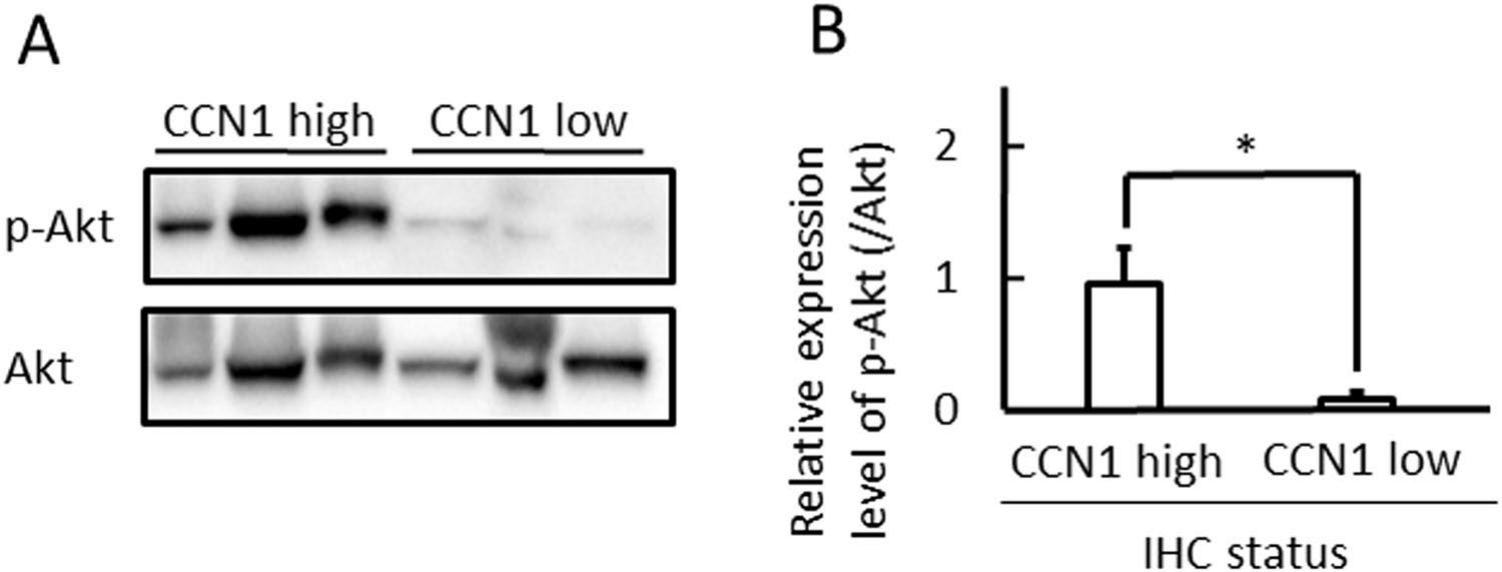
PI3K/AKT/mTOR pathway is upregulated in CCN1 high expression group. (**A**) Immunoblot analysis of levels of p-Akt and Akt total protein in CCN1 high and low expression cases. (**B**) Quantification of data from panel A. Statistical significance was calculated by the Student’s *t*-test. *p < 0.05. Data are shown as the mean ± SEM. Full-length blots are presented in Supplementary Figure S2.

To investigate the relationship between CCN1 and PI3K signalling in glioblastoma cases, we evaluated p-Akt levels and p85 protein expression using western blotting (n = 25) and immunohistochemistry (n = 70), respectively. Western blotting demonstrated that the CCN1 high expression group showed high p-Akt at Thr308 compared with the CCN1 low expression group (Fig. 5A and B; *p* < 0.05). We also evaluated p85 protein expression in 70 glioblastoma patients using the immunohistochemical scoring system as previously described^26^. However, there was no difference in p85 expression between the CCN1 high and low expression groups (Supplementary Fig. S1A,C, and D). This result corresponds to data from cBioPortal^27, 28^ (Supplementary Fig. S1B).

### Overexpression of either CCN1 or *PIK3R1Met326Ile* promoted glioma cell invasion *in vitro*. 

**Figure 6:**
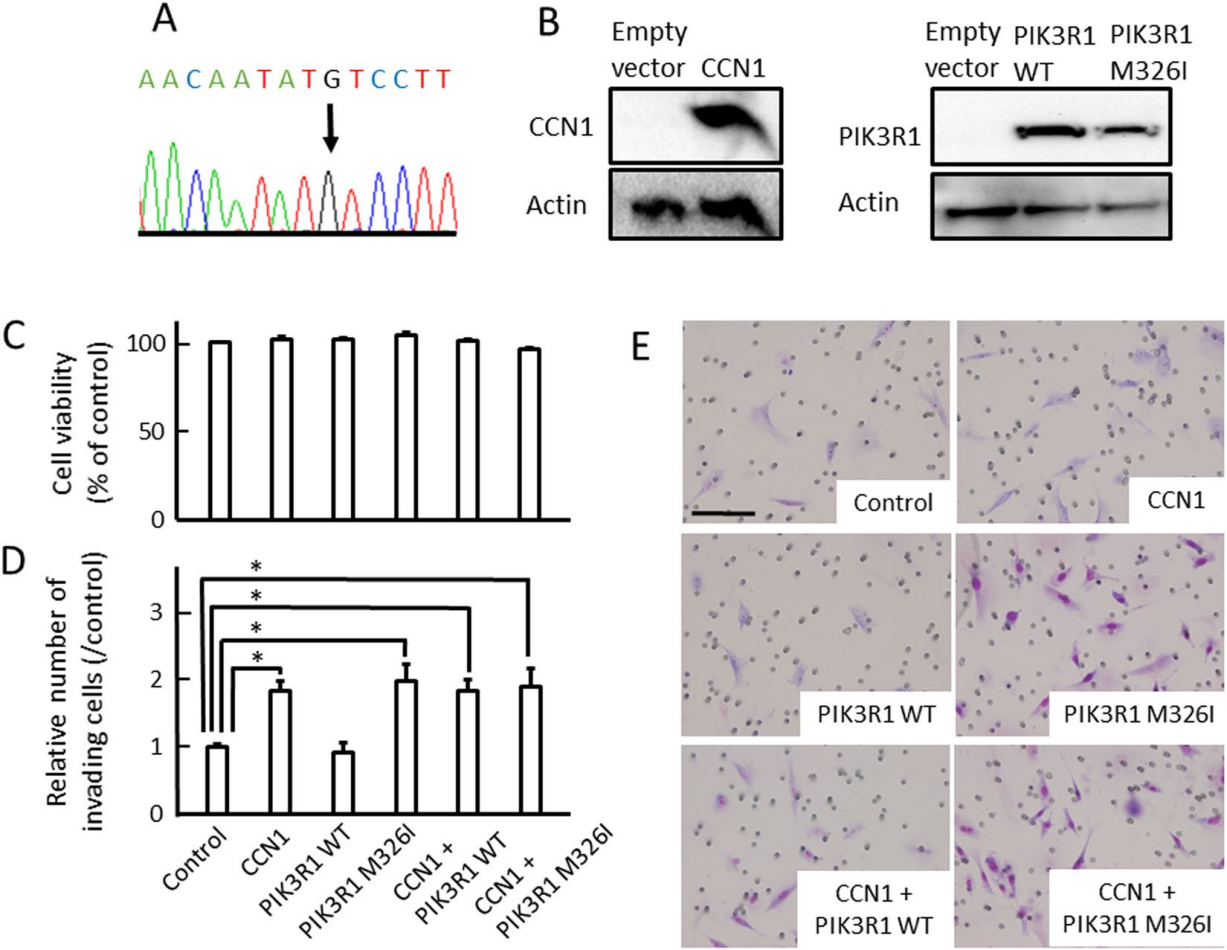
The role of PIK3R1Met326Ile on cell process. (A) Sequencing of PIK3R1 in U87MG. (**B**) Western blotting of CCN1 and PIK3R1 protein in U87MG cells transfected with the indicated plasmids. Full-length blots are presented in Supplementary Figure S2. (**C**) WST-1 assays for cell proliferation in U87MG cells transfected with the indicated plasmids (n = 5). (**D**,**E**) Migration assays in U87MG cells transfected with the indicated plasmids (n = 3). Quantification of migrated cells are shown on the graph in **D**. Representative images of each cell transfection group are shown in **E**. Statistical significance was calculated by ANOVA with Dunnett’s post hoc test. *p < 0.05. Data in C and D are shown as the mean ± SEM. Scale bar = 100 μm.

Finally, to address the biological relevance of CCN1 and *PIK3R1Met326Ile* expression, we evaluated the role of CCN1 and the *PIK3R1Met326Ile* mutation on cellular activities using WST-1 and invasion assays. Plasmids expressing CCN1, wild-type PIK3R1, mutant PIK3R1, or a combination of these plasmids were transfected into U87MG glioma cells, which carry low CCN1 expression^29^, no *PIK3R1* somatic mutations^30^ and PIK3R1Met326Ile germline mutation (Fig. 6A). Western blot analysis confirmed that overexpression vectors induced detectable levels of CCN1, PIK3R1 and PIK3R1 mutant (Fig. 6B). WST-1 assay showed that overexpression of CCN1, PIK3R1 or PIK3R1 mutant had no effect on cell proliferation (Fig. 6C). In contrast, invasion assays showed that CCN1 and PIK3R1 mutant both significantly increased cell invasion compared to the control (Fig. 6D and E; *p* < 0.05). Furthermore, the combination of CCN1 together with wild-type PIK3R1, and CCN1 with PIK3R1 mutant showed enhanced cell motility; however, there was no difference between these groups.

## Discussion

In this study, high expression of CCN1 was related to cCCN1 expression and highly germline mutation of *PIK3R1Met326Ile*, and *PIK3R1Met326Ile* was found to be a prognostic factor in glioblastoma patients.

CCN1 is widely expressed in several tumor types and has been linked to a poor prognosis in cancer patients, including those with glioma^15, 16, 31, 32^. Previously, CCN1 expression was revealed to be an independent prognostic factor for glioblastoma patients^11^. Full-length CCN1 (flCCN1) is a 42-kDa protein, which is converted into cCCN1 by secreted plasmin in the extracellular matrix, resulting in two peptide fragments: the 28-kDa fragment corresponds to the NH2-terminal of CCN1 and the 21-kDa fragment corresponds to the COOH-terminal^20, 33^. Because the NH2-terminal fragment (28-kDa) promotes cell migration in breast cancer^20^, this fragment was analyzed by western blotting, and cCCN1 in western blotting was significantly correlated with CCN1 in immunohistochemical staining.

Activation of the PI3K pathway, which comprises PIK3R1, is evident in many cancers, including glioblastoma. Reports suggest that activation of PI3K signaling with overexpression of Akt or loss of PTEN leads to gliomagenesis^34–36^, while Bai *et al*. showed that PI3K signaling was one of the three important pathways in *IDH1*-mutant glioma progression^37^. To date, somatic mutations of *PIK3R1* in glioma have been well-researched. Quayle *et al*. found that several types of somatic mutation in the iSH2 domain of *PIK3R1* promoted gliomagenesis^30^. However, germline mutations in *PIK3R1* were not fully elucidated. A single nucleotide polymorphism in codon 326 of *PIK3R1*, comprising Met substituted by Ile and located near to the NH2-terminal SH2 domain coding region, is known as a germline mutation. Only a few reports have been published on *PIK3R1Met326Ile* mutations in cancer. Li *et al*. showed that individuals carrying one or two copies of the 326Ile variant had a 47% increased risk of colon cancer compared with Met/Met^38^. Hasheminasab *et al*. reported that cancer patients with the *PIK3R1Met326Ile* variation were predisposed to skin rashes induced by epidermal growth factor receptor (EGFR) inhibitors, but tended to have better OS^39^. The *PIK3R1Met326Ile* mutation was found to be a novel genetic biomarker in gastric cancer, and patients with this germline mutation displayed higher sensitivity to GSK2636771, a PI3Kβ-selective inhibitor^40^. Kim J. H. *et al*. reported that in metastatic renal cell carcinoma, there was a tendency for poorer responses to vascular endothelial growth factor receptor (VEGFR) tyrosine kinase inhibitors in patients harboring *PIK3R1Met326Ile* germline mutations compared with wild-type *PIK3R1* ^41^.

Functional studies of the *PIK3R1Met326Ile* mutation are scarce. Almind *et al*. reported that the *PIK3R1Met326Ile* mutation was associated with a lower level of cellular p85α protein in diabetes mellitus^42^, but it had no impact on PI3K activity^43^. Cheung also found that the *PIK3R1Met326Ile* mutation had no effect on Ba/F3 cell survival or Akt phosphorylation^44^. However, in gastric cancer, *PIK3R1Met326Ile* was associated with increased p85α expression^40^. In this study, we found that U87MG cells transfected with plasmids expressing either CCN1 or PIK3R1Met326Ile showed significantly increased invasion ability compared with control cells. Some researchers have already reported that CCN1 is involved in glioma invasion^45, 46^. Also, somatic mutation of PIK3R1 is known to be related with the increasing of cell proliferations and invasion in cancer, however, the role of germline mutation of PIK3R1 have not been fully revealed^18^.

Some germline mutations are known to participate in certain familial gliomas; however, the relationship between germline mutations and tumorigenesis or prognosis remains unclear. As described above, *PIK3R1Met326Ile* germline mutations are related to chemosensitivity in several cancers, including EGFR inhibitors, a PI3Kβ-isoform selective inhibitor, and tyrosine-kinase inhibitors^39, 40^. Recently, clinical trials have been conducted to identify therapeutic targets for cancers based on specific genetic alterations^24, 47^, and our results may be highly relevant to these trials.

Limitations of this study is the data were from a single center. Therefore, further investigations using large cohorts, such as TCGA data, are necessary to demonstrate that the key findings are applicable to other centers. Our results also showed the significance of *PIK3R1Met326Ile* in glioblastoma but not in lower grade glioma. One reason may be that lower grade glioma comprises many types of glioma. Our study included 41 astrocytoma and 19 oligodendroglioma cases, but the patients were diagnosed based on histology rather than molecular confirmations. This might affect the result, and further studies should include lower grade glioma cases diagnosed by molecular patterns.

In conclusion, high expression of CCN1 by immunohistochemical staining was significantly correlated with cCCN1 on western blotting, and the *PIK3R1Met326Ile* germline mutation existed at a high rate in the CCN1 high expression group. In addition, the *PIK3R1Met326Ile* germline mutation was significantly correlated with the prognosis of glioblastoma patients. These results may contribute toward the development of novel therapeutic strategies based on molecular alterations.

## Material and Methods

### Patients

One hundred forty-seven glioma specimens were excised from 80 glioblastoma patients and 67 lower grade glioma patients treated at the Okayama University Hospital between 2006 and 2017. The study (No. 1603–070) was approved by the ethical committee of the Okayama University Graduate School of Medicine, Dentistry and Pharmaceutical Sciences, Okayama, Japan and carried out in accordance with the approved guidelines. All patients provided informed written consent.

### Immunohistochemistry

For immunohistochemistry, all specimens were fixed in 10% formaldehyde solution, embedded in paraffin blocks, and then sliced into 4.5-µm sections. Paraffin-embedded sections were deparaffinized, epitope-retrieved with an autoclave, and blocked with 5% normal goat serum in phosphate-buffered saline (PBS). Slices were incubated with primary antibodies (anti-CYR61, 1:100, Novus Biologicals, Littleton, CO, USA; anti-PIK3R1, 1:100, Sigma-Aldrich, St Louis, MO, USA). The Dako Cytomation Envision + System-HRP Kit was then used in accordance with the manufacturer’s protocol (DakoCytomation, Carpentaria, CA, USA). After washing in PBS, the sections were counterstained with hematoxylin. Immunohistochemistry samples were observed with a BZ-8100 fluorescence microscope (Keyence, Osaka, Japan). CCN1 protein is expressed in the nucleus, cytoplasm and membrane, and also extracellular areas. The amount of CCN1 immunoreactivity was measured according to the immunoreactive intensity and the estimated extent of the immunopositive area, expressed as a percentage of the total (0–14%, low; 15–100%, high). The cut-off value of CCN1 was previously reported to be 15% of the immunopositive area^11^.

### Western blotting

For immunoblot analysis, cells were lysed in RIPA buffer (Cell Signaling Technology, Danvers, MA, USA) containing phenylmethanesulfonyl fluoride (PMSF, Cell Signaling Technology), and then sonicated. Samples were loaded for sodium dodecyl sulfate (SDS)-polyacrylamide gel electrophoresis and the separated proteins were blotted onto PVDF membranes. After blocking in 5% skimmed milk, the membranes were incubated overnight with primary antibodies at 4 °C. The membranes were washed with Tris-buffered saline-Tween 20 (TBST), incubated with secondary antibodies at room temperature for 1 h, and rinsed with TBST. HRP signals were visualised using an ECL prime western blotting detection system (GE Healthcare, Little Chalfont, UK) and detected by VersaDoc (Bio-Rad, Hercules, CA, USA). For the immunoblot analysis, the primary antibodies were anti-CYR61 (1:300), p-Akt (1:1000, Cell Signaling Technology), Akt (1:1000, Cell Signaling Technology) and β-actin (1:5000, Sigma-Aldrich), with anti-mouse and anti-rabbit IgG HRP-linked secondary antibodies (both 1:5000, Cell Signaling Technology).

### DNA extraction

The QIAamp DNA Micro kit (QIAGEN, Santa Clarita, CA, USA) and QIAamp DNA FFPE Tissue kit (QIAGEN) were used according to the manufacture’s protocol to extract genomic DNA from freshly frozen tumor tissue, FFPE tissue and blood.

### Next-generation sequencing

Each genomic DNA sample from tissue specimens was subjected to target amplification and library preparation for next-generation sequencing analysis using a Haloplex Cancer Panel according to the manufacturer’s protocol (Agilent Technologies, Santa Clara, CA, USA). The target enrichment library pool was sequenced using Miseq (Illumina, San Diego, CA, USA). Sequence data were aligned, analyzed, and visualized using SureCall 2.0 software (Agilent Technologies).

### Sanger sequencing

Oligo primers were designed for amplifying a target lesion of *PIK3R1* (forward; 5′-ATTGCATGGAATTGTGAACTAATGC-3′, reverse; 5′-TGTTCTTAGGCAGTGCCACTTCA-3′). The Pfu DNA polymerase (Agilent technologies) and optimized thermal conditions were used for polymerase chain reaction (PCR). The PCR products were analyzed using the Big Dye Terminator kit (Applied Biosystems, CA, USA) and an ABI 3130xl capillary sequencer (Applied Biosystems).

### Cell culture

The U87MG cell line was purchased from DS Pharma Biomedical (Osaka, Japan) in September 2016 and authenticated by Promega (Madison, WI, USA) via STR profiling in December 2016. U87MG cells were cultured in Dulbecco’s Modified Eagle’s Medium (DMEM) with 10% fatal bovine serum, 100 U of penicillin, and 0.1 mg mL^−1^ of streptomycin.

### Plasmids

The *PIK3R1* plasmids were purchased from GeneCopoeia (Rockville, MD, USA). Site-directed mutagenesis was performed using the GENEART Site-Directed Mutagenesis System (Invitrogen, Carlsbad, CA, USA) to produce the *PIK3R1Met326Ile* plasmid. The CCN1 plasmid was provided by Dr. H. Phillip Koeffler, Cedars-Sinai Medical Center, UCLA School of Medicine (LA, USA). Cells were transfected with plasmids using Lipofectamine^®^ 3000 (Invitrogen) according to the manufacturer’s instructions.

### Water soluble tetrazolium-1 (WST-1) assay

For analyses of glioma cell proliferation, the WST-1 assay was performed according to the manufacturer’s protocol (Roche, Mannheim, Germany).

### Invasion assay

Invasion assays were performed using a BioCoat Matrigel invasion chamber (BD Bioscience, Franklin Lakes, NJ, USA) according to the manufacturer’s instructions. In brief, 2 × 10^5^ cells were seeded in DMEM with 0.1% FBS in the upper chamber. The lower chamber was filled with DMEM with 10% FBS. After 24 h incubation, the filters of inserts were fixed with methanol and stained with Giemsa solution. The number of invading cells on the lower surface of the filter was counted.

### Statistical analysis

Protein expression was assessed with the Mann-Whitney U test. The frequency of each mutation was analysed using a paired t-test or Fisher’s exact test. Kaplan-Meier curves were compared using the log-rank test. The results of WST-1 assay and invasion assay were calculated by ANOVA with Dunnett’s post hoc test. Statistical analyses were performed using the SPSS statistical software (version 20; SPSS, Chicago, IL, USA). *P*-values < 0.05 were considered to denote statistically significant differences.

## Electronic supplementary material

Supplementary Information

## References

[1] Stupp R (2005). Radiotherapy plus concomitant and adjuvant temozolomide for glioblastoma. N Engl J Med.

[2] Chinot OL (2014). Bevacizumab plus radiotherapy-temozolomide for newly diagnosed glioblastoma. N Engl J Med.

[3] Gilbert MR (2014). A randomized trial of bevacizumab for newly diagnosed glioblastoma. N Engl J Med.

[4] Bedard PL, Hansen AR, Ratain MJ, Siu LL (2013). Tumour heterogeneity in the clinic. Nature.

[5] Verhaak RG (2010). Integrated genomic analysis identifies clinically relevant subtypes of glioblastoma characterized by abnormalities in PDGFRA, IDH1, EGFR, and NF1. Cancer Cell.

[6] Brennan CW (2013). The somatic genomic landscape of glioblastoma. Cell.

[7] Brell M (2005). Prognostic significance of O6-methylguanine-DNA methyltransferase determined by promoter hypermethylation and immunohistochemical expression in anaplastic gliomas. Clin Cancer Res.

[8] Hegi ME (2005). MGMT gene silencing and benefit from temozolomide in glioblastoma. N Engl J Med.

[9] Parsons DW (2008). An integrated genomic analysis of human glioblastoma multiforme. Science.

[10] Yan H (2009). IDH1 and IDH2 mutations in gliomas. N Engl J Med.

[11] Ishida J (2015). Evaluation of extracellular matrix protein CCN1 as a prognostic factor for glioblastoma. Brain Tumor Pathol.

[12] Walsh CT (2008). Thrombin receptor and RhoA mediate cell proliferation through integrins and cysteine-rich protein 61. FASEB J.

[13] O’Brien TP, Yang GP, Sanders L, Lau LF (1990). Expression of cyr61, a growth factor-inducible immediate-early gene. Mol Cell Biol.

[14] Babic AM, Kireeva ML, Kolesnikova TV, Lau LF (1998). CYR61, a product of a growth factor-inducible immediate early gene, promotes angiogenesis and tumor growth. Proc Natl Acad Sci U S A.

[15] Xie D (2001). Breast cancer. Cyr61 is overexpressed, estrogen-inducible, and associated with more advanced disease. J Biol Chem.

[16] Xie JJ (2011). Expression of cysteine-rich 61 is correlated with poor prognosis in patients with esophageal squamous cell carcinoma. Eur J Surg Oncol.

[17] Costa C, Engelman JA (2014). The double life of p85. Cancer Cell.

[18] Cheung LW (2014). Naturally occurring neomorphic PIK3R1 mutations activate the MAPK pathway, dictating therapeutic response to MAPK pathway inhibitors. Cancer Cell.

[19] Robertson LB (2010). Survey of familial glioma and role of germline p16INK4A/p14ARF and p53 mutation. Fam Cancer.

[20] Pendurthi UR, Tran TT, Post M, Rao LV (2005). Proteolysis of CCN1 by plasmin: functional implications. Cancer Res.

[21] Guillon-Munos A (2011). Kallikrein-related peptidase 12 hydrolyzes matricellular proteins of the CCN family and modifies interactions of CCN1 and CCN5 with growth factors. J Biol Chem.

[22] Paugh BS (2010). Integrated molecular genetic profiling of pediatric high-grade gliomas reveals key differences with the adult disease. J Clin Oncol.

[23] Jones, C. *et al*. Pediatric high-grade glioma: biologically and clinically in need of new thinking. *Neuro Oncol* (2016). 10.1093/neuonc/now101 PMC5464243 27282398

[24] Project., I. C. G. C. P. T. (2016). Recurrent MET fusion genes represent a drug target in pediatric glioblastoma. Nat Med.

[25] Kameda, M. *et al*. Congenital Glioblastoma with Distinct Clinical and Molecular Characteristics: Case Reports and a Literature Review. *World Neurosurg* (2017). 10.1016/j.wneu.2017.02.026 28214639

[26] Cizkova M (2013). PIK3R1 underexpression is an independent prognostic marker in breast cancer. BMC Cancer.

[27] Gao J (2013). Integrative analysis of complex cancer genomics and clinical profiles using the cBioPortal. Sci Signal.

[28] Cerami E (2012). The cBio cancer genomics portal: an open platform for exploring multidimensional cancer genomics data. Cancer Discov.

[29] Kurozumi K (2008). Oncolytic HSV-1 infection of tumors induces angiogenesis and upregulates CYR61. Mol Ther.

[30] Quayle SN (2012). Somatic mutations of PIK3R1 promote gliomagenesis. PLoS One.

[31] Xie D (2004). Levels of expression of CYR61 and CTGF are prognostic for tumor progression and survival of individuals with gliomas. Clin Cancer Res.

[32] Sabile AA (2012). Cyr61 expression in osteosarcoma indicates poor prognosis and promotes intratibial growth and lung metastasis in mice. J Bone Miner Res.

[33] Moon HG (2014). CCN1 secretion and cleavage regulate the lung epithelial cell functions after cigarette smoke. Am J Physiol Lung Cell Mol Physiol.

[34] Holland EC (2000). Combined activation of Ras and Akt in neural progenitors induces glioblastoma formation in mice. Nat Genet.

[35] Zheng H (2008). p53 and Pten control neural and glioma stem/progenitor cell renewal and differentiation. Nature.

[36] Sonoda Y (2001). Akt pathway activation converts anaplastic astrocytoma to glioblastoma multiforme in a human astrocyte model of glioma. Cancer Res.

[37] Bai H (2016). Integrated genomic characterization of IDH1-mutant glioma malignant progression. Nat Genet.

[38] Li L, Plummer SJ, Thompson CL, Tucker TC, Casey G (2008). Association between phosphatidylinositol 3-kinase regulatory subunit p85alpha Met326Ile genetic polymorphism and colon cancer risk. Clin Cancer Res.

[39] Hasheminasab SM (2015). High-throughput screening identified inherited genetic variations in the EGFR pathway contributing to skin toxicity of EGFR inhibitors. Pharmacogenomics.

[40] Kim, C. *et al*. Whole-exome sequencing of gastric cancer identifies germline PIK3R1 variant as a novel genetic biomarker for a PI3K beta-isoform selective inhibitor, GSK2636771. In: Proceedings of the American Association for Cancer Research; 2015 Apr 18–22; Philadelphia, PA: AACR; 2015. Abstract nr 4694.

[41] Kim, J. H. *et al*. Novel biomarkers for VEGFR inhibitors in metastatic renal cell carcinoma: BIM expression, and germline polymorphisms of BIM and PIK3R1. In: Proceedings of the American Association for Cancer Research; 2016 Apr 16–20; New Orleans, LA: AACR; 2016. Abstract nr 430.

[42] Almind K (2002). Characterization of the Met326Ile variant of phosphatidylinositol 3-kinase p85alpha. Proc Natl Acad Sci U S A.

[43] Baynes KC (2000). Natural variants of human p85 alpha phosphoinositide 3-kinase in severe insulin resistance: a novel variant with impaired insulin-stimulated lipid kinase activity. Diabetologia.

[44] Cheung LW (2011). High frequency of PIK3R1 and PIK3R2 mutations in endometrial cancer elucidates a novel mechanism for regulation of PTEN protein stability. Cancer Discov.

[45] Young N, Pearl DK, Van Brocklyn JR (2009). Sphingosine-1-phosphate regulates glioblastoma cell invasiveness through the urokinase plasminogen activator system and CCN1/Cyr61. Mol Cancer Res.

[46] Haseley A (2012). Extracellular matrix protein CCN1 limits oncolytic efficacy in glioma. Cancer Res.

[47] Worst BC (2016). Next-generation personalised medicine for high-risk paediatric cancer patients - The INFORM pilot study. Eur J Cancer.

