# Supplementary material for: PIK3R1Met326Ile germline mutation correlates with cysteine-rich protein 61 expression and poor prognosis in glioblastoma
Source: Sci Rep. 2017 Aug 7;7:7391. doi: 10.1038/s41598-017-07745-0 (PMC5547066; doi:10.1038/s41598-017-07745-0)
Supplement: Supplementary file 1 — Supplementary Information [file 41598_2017_7745_MOESM1_ESM.pdf]

***PIK3R1Met326Ile* germline mutation correlates with cysteine-rich protein 61 expression and poor prognosis in glioblastoma**

Yoshihiro Otani 1), Joji Ishida 1), Kazuhiko Kurozumi 1)*, Tetsuo Oka 1), Toshihiko Shimizu 1), Yusuke Tomita 1), Yasuhiko Hattori 1), Atsuhito Uneda 1), Yuji Matsumoto 1), Hiroyuki Michiue 2), Shuta Tomida 3)4), Takehiro Matsubara 3), Tomotsugu Ichikawa 1), Isao Date 1)

1) Department of Neurological Surgery, Okayama University Graduate School of Medicine, Dentistry, and Pharmaceutical Sciences, Okayama, Japan

2) Department of Physiology, Okayama University Graduate School of Medicine, Dentistry, and Pharmaceutical Sciences, Okayama, Japan

3) Okayama University Hospital Biobank, Okayama University Hospital, Okayama, Japan

4) Department of Biobank, Graduate School of Medicine, Dentistry and Pharmaceutical Sciences, Okayama University, Okayama, Japan

*Correspondence: Kazuhiko Kurozumi

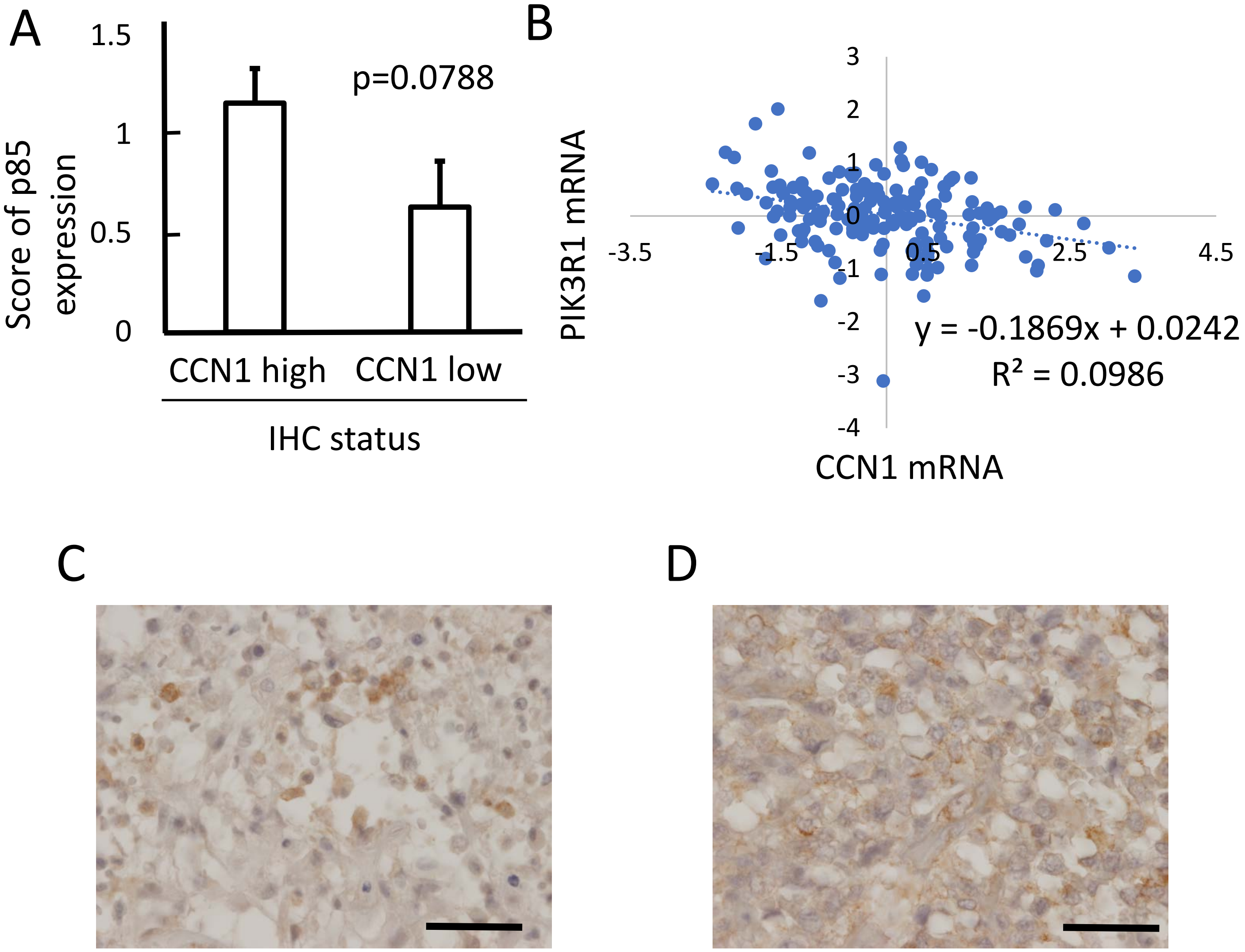

Supplementary Figure S1: Immunohistochemistry of p85 expression in CCN1 high and low expression groups.

(A) Quantification of p85 expression determined by immunohistochemistry in the CCN1 high and low expression groups.

(B) Data from cBioPortal showed that there was no significant relationship between CCN1 mRNA and PIK3R1 mRNA expression.

(C, D) Representative immunohistochemical staining of PIK3R1 expression in glioblastoma multiforme patients. C: CCN1 high expression group, D: CCN1 low expression group. Scale bar = 50 μm. The statistical significance was calculated by the Student's t-test. *$p < 0.05$. Data are shown as the mean $\pm$ SEM.

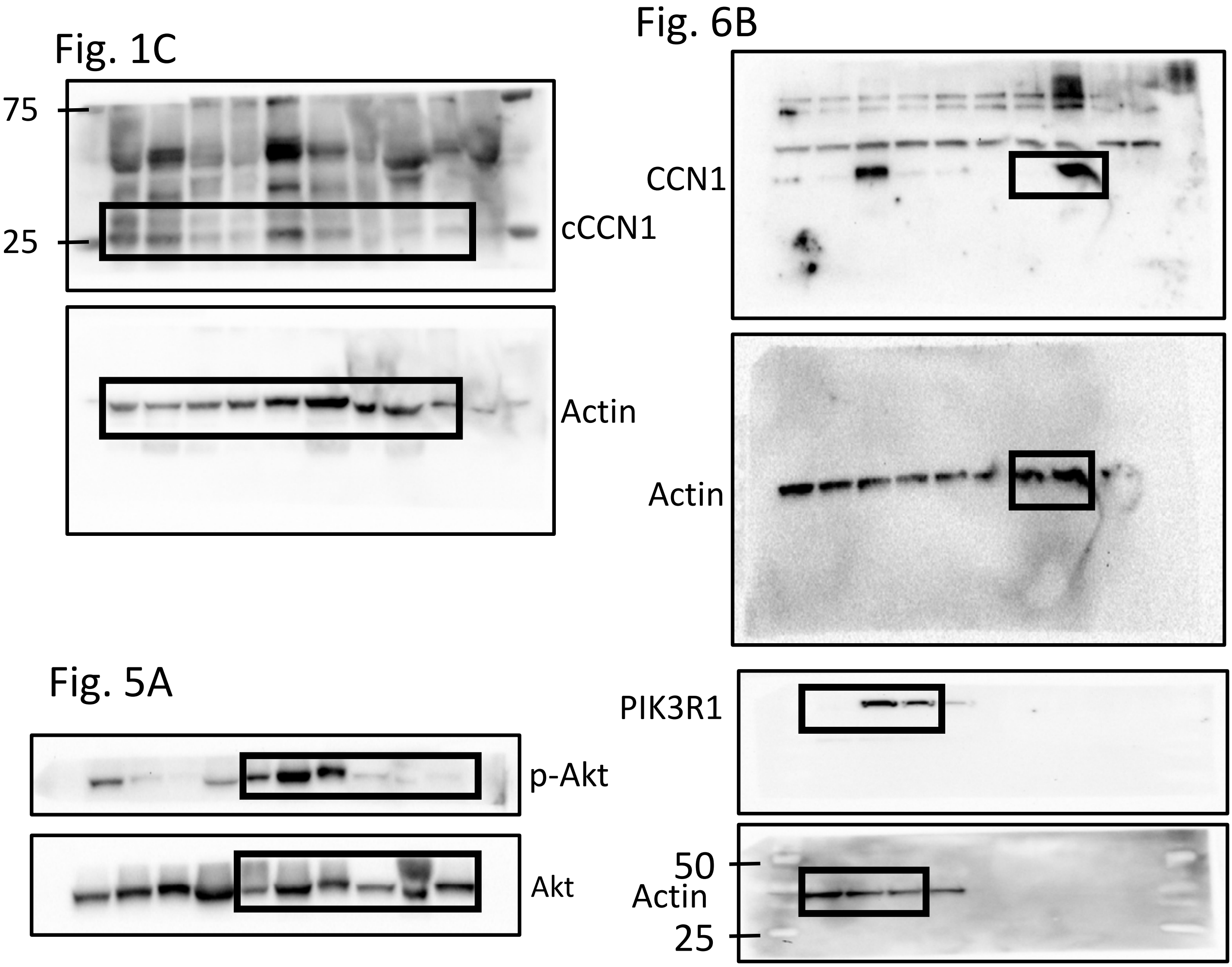

Supplementary Figure S2: Full scans of the immunoblots shown in the figure. Boxes indicated parts used in the figure and numbers indicated molecular weights.

Supplementary Table S1

Clinical and genetic characteristics of the oligodendroglioma study cohort.

Characteristic	Total cases (N=19)	CCN1 high (N=6)	CCN1 low (N=13)
Age - year			
Median	51	56	51
Range	21-82	21-68	23-82
Sex - no.(%)			
Male	11 (58.0)	3 (50.0)	8 (62.0)
Female	8 (42.0)	3 50.0)	5 (48.0)
Grade – no.(%)			
Grade II	6 (31.6)	2 (33.3)	4 (30.8)
Grade III	13 (88.4)	4 (66.7)	9 (69.2)
Surgical status – no.(%)*			
Biopsy or partial resection	13 (72.2)	5 (83.3)	8 (66.7)
Gross total resection	5 (27.8)	1 (16.7)	4 (33.3)
CCN1 expression– no.(%)			
High	6 (31.6)		
Low	13 (68.4)		
PIK3R1Met326Ile mutation – no.(%)			
Positive	2 (10.5)	1 (16.7)	1 (7.7)
Negative	17 (89.5)	5 (83.3)	12 (92.3)

*We could not investigate the extension of resection of one patient.

Supplementary Table S2

Univariate analysis of prognostic factors for progression-free survival and overall survival in astrocytoma patients.

	No. of patients (%)	*p* value	
		PFS	OS
Age (years)			
≧65	12 (30.0)	0.0432	0.0010
< 65	30 (70.0)		
Sex			
Male	24 (57.1)	0.677	0.7251
Female	18 (42.9)		
Grade			
II	6 (14.3)	0.8557	0.4540
III	36 (85.7)		
Surgical status*			
Biopsy or partial resection	27 (65.9)	0.1675	0.5885
Gross total resection	14 (34.1)		
CCN1 expression			
High	19 (45.2)	0.3663	0.6570
Low	23 (54.8)		
PIK3R1Met326Ile			
Positive	8 (19.0)	0.9114	0.7809
Negative	34 (81.0)		

PFS = progression free survival; OS = overall survival

* We could not investigate the extension of resection of one patient.

Supplementary Table S3

Univariate analysis of prognostic factors for progression-free survival and overall survival in oligodendroglioma patients.

	No. of patients (%)	*p* value	
		PFS	OS
Age (years)			
≧65	4 (21.1)	0.0018	0.2864
< 65	15 (78.9)		
Sex			
Male	11 (57.9)	0.7733	0.7053
Female	8 (42.1)		
Grade			
II	6 (31.6)	0.3588	N/A
III	13 (68.4)		
Surgical status*			
Biopsy or partial resection	13 (31.6)	0.8466	N/A
Gross total resection	5 (68.4)		
CCN1 expression			
High	6 (31.6)	0.7824	N/A
Low	13 (68.4)		
PIK3R1Met326Ile			
Positive	2 (10.5)	N/A	N/A
Negative	17 (89.5)		

PFS = progression free survival; OS = overall survival; N/A = not available

*We could not investigate the extension of resection of one patient.
